# Advanced Paternal Age and Future Generations

**DOI:** 10.3389/fendo.2022.897101

**Published:** 2022-06-09

**Authors:** Peter T. K. Chan, Bernard Robaire

**Affiliations:** ^1^Department of Urology, McGill University Health Centre, Montreal, QC, Canada; ^2^Department of Pharmacology & Therapeutics, McGill University, Montreal, QC, Canada; ^3^Department of Obstetrics & Gynecology, McGill University, Montreal, QC, Canada

**Keywords:** spermatozoa, oxidative stress, animal models, artificial reproduction technologies, progeny outcome

## Abstract

Paternal age at conception has been increasing. In this review, we first present the results from the major mammalian animal models used to establish that increasing paternal age does affect progeny outcome. These models provide several major advantages including the possibility to assess multi- transgenerational effects of paternal age on progeny in a relatively short time window. We then present the clinical observations relating advanced paternal age to fertility and effects on offspring with respect to perinatal health, cancer risk, genetic diseases, and neurodevelopmental effects. An overview of the potential mechanism operating in altering germ cells in advanced age is presented. This is followed by an analysis of the current state of management of reproductive risks associated with advanced paternal age. The numerous challenges associated with developing effective, practical strategies to mitigate the impact of advanced paternal age are outlined along with an approach on how to move forward with this important clinical quandary.

## Introduction

We are witnessing the progressive increase of paternal age at conception. The birth rate among 35 to 49 year old American men in 2015 was 69.1 per thousand compared with 42.8 per thousand in 1980 (1). Other countries have reported a similar trend (2) that appears to be consistent across all races, ethnicities, regions and level of education (3). While controversies exist, a preponderance of evidence from recent scientific literature affirms a negative impact of advanced paternal aging on reproductive health. In this review we will begin by discussing the role of animal models as a valuable research tools to study the effects of paternal aging. After presenting how advanced paternal age impacts the fertility status of men, reproductive outcomes and offspring health, we will provide an opinionated analysis on the challenges faced by healthcare providers and health authorities in the development and implementation of practical strategies designed to reduce or mitigate the negative impact of advanced paternal age from a public health point of view.

## Animal Models for Studying Paternal Aging

A wide range of animal models ranging from insects to worms, birds, fish and mammals have been used to investigate the effects of paternal aging on male reproduction function. However, rodent models have become the predominant species for determining the cellular and molecular changes with aging that occur in the testis and epididymis (4–6). Outbred rodents are often used in drug testing or environmental exposure studies so to increase the genetic variability in the population. However, inbred rodents are preferred for aging studies that focus specifically on the mechanistic pathways in question. A potential limitation is that several pathologies associated with aging including pituitary, adrenal or testis tumors may complicate result interpretation. An ideal animal model should be long-lived and free from the systemic aging-related diseases, while maintaining other reproductive changes that emulate those in aging men.

### Mouse Models

Studies using mouse models that lack any known or induced mutations have demonstrated a quantitative reduction in spermatozoa with increased age. Testicular architecture reveals changes in tubule segments with impaired spermatogenesis, increased number of vacuoles in Sertoli and germ cells, a thinning of the seminiferous epithelia, and a reduction in the number of spermatocytes and spermatids (7, 8). An increase in age-related germ cell mutations has also been reported (9).

Several inbred strains of mice, such as the senescence-accelerated mouse (10, 11), and transgenic mice, such as Klotho mice (12, 13), have been developed to model accelerated aging in humans. These mice exhibit defects in a wide range of organs (e.g., vessels, lungs, kidney, brain, skin and testes), and thus are poor models to study aging of male germ cells as many interfering systems could be operant. However, an advantage of the mouse model is the feasibility for genetic manipulations for both over-expressed and knocked out genes, and consequently allows for studies investigating mechanisms involved in aging. Mice overexpressing catalase have reduced ROS and do not exhibit the age-dependent loss of spermatozoa, do show aging-associated loss in testicular germ and Sertoli cells, and show reduced 8-oxodG lesions in spermatozoa (14). In contrast, null mutations for superoxide dismutase show exacerbated age-induced damage in both the testis histology and spermatozoa quality (15).

### Rat Models

With its long lifespan and relatively free of age-related pathologies including tumors and obesity, the Brown Norway (BN) rat is a highly robust model for the study of male reproductive aging (16–19). Striking age-related changes in the seminiferous tubules (16), Leydig cells (5) and epididymides (20) of these animals have been reported. Several genes in the testis (Leydig and germ cells) and in the epididymis have altered expression as a function of aging (21–23). With advancing age, Sertoli cells, the niche-forming “nurse” cells that surround the germ cells and ensure their normal development, display anomalies in the structure of the endoplasmic reticulum and nuclei; large intracellular spaces are observed between Sertoli cells, rather than the normally embedded germ cells (24). Genes and proteins associated with the formation of the blood-testis barrier decline prior to the barrier becoming “leaky” (25). Effects of aging are also seen in the hypothalamic-pituitary function (17, 26). Importantly, the changes seen in testis and hypothalamic-pituitary functions in the BN rat with age reflect those reported in aging men (27, 28).

Mating of male BN rats of increasing age (3–24 months) to young females result in an increase in pre-implantation loss, a decrease in the average fetal weight, and an increase in neonatal deaths (29). Together, these results show that the quality of spermatozoa decreases as BN male rats age. The basis for these age-related declines in reproductive function remains unclear. In isolated populations of testicular germ cells, the expression of a number of genes is affected during ageing (21, 30). The findings of a large increase in sperm with abnormal flagellar midpieces, decrease in the percentage of motile spermatozoa and elevation of immature spermatozoa retaining their cystoplasmic droplets in the cauda epididymides of old rats suggests a defective spermatozoa formation in aging testes (31) and impaired epididymal function in supporting sperm maturation. We reported previously aging related increase in basal sperm chromatin damage with age (32) which suggest an accumulation of DNA damage and/or mutations in the germ line that may contribute to adverse health outcomes of their offspring.

### Advantages and Limitations of Animal Models Over Human Studies

Animal models have clear advantages for control over the homogeneity of the genetic pool, for conducting controlled mating studies and for access to all cells of the reproductive system for analyses. Indeed, studies using animal models have unequivocally established that increased paternal age is associated with decreased sperm number and chromatin quality, and adverse progeny outcome. For therapeutic and interventional studies, animal models allow for control of confounders seen often in human studies such as obesity, diet, exposure to toxins and the age of female mates. Finally, it is possible to assess multi- transgenerational effects of paternal age on progeny in a relatively short time window.

Aging studies with animal are not without limitations. The relatively shorter lifespan of rodents limits the wide range of environmental exposures to chemicals that can impinge on sperm function and production. Further, quantitatively and qualitatively, men are far less effective at producing sperm per gram of testis (33), possibly due to postural position and bypass of temperature regulation for optimal spermatogenesis. Finally, although the number of genes in man and rodents are similar, the human genome contains far more non-genomic DNA that likely plays a role in epigenetic regulation of germ cell functions (34). Thus, a comprehensive understanding of how paternal age affects both the genome and epigenome of spermatozoa, and the consequences of these effects will require complementary animal and human studies.

## Impact of Advanced Paternal Age in Men and on Their Progeny

### Impact of Advanced Paternal Age on Male Fertility Status

Various studies have indicated an age-related decline of conventional semen parameters including semen volume, total sperm count, motility and morphology (35). Not surprisingly, natural fertility rates decline as men age, as demonstrated by a survey that conception at 1yr is 30% less for men >40yrs versus those <30yrs (36). Similar findings were reported by Hassan and Killick (37). Natural conceptions with men >35yrs were found to be 1.26 times more likely to miscarry than those with men <35yrs (38). In a retrospective cohort study from 1989–2005, pregnancies sired by father >45yrs showed a 48% increased risk of late stillbirth, a 19% increased risk of low birth weight, a 13% increased risk of preterm birth and a 29% increased risk of very preterm birth (39).

### Impact of Advanced Paternal Age on Assisted Reproductive Outcomes

Advanced paternal age has been associated with various adverse outcomes with assisted reproductive technologies (ARTs) including poor embryo quality, increased miscarriage rates, reduced fertilization, implantation, pregnancy, and live birth rates (40–48). Inconsistency and conflicting data exist (49–51) likely due to the results of confounders and bias in the design of the studies, small sample size, retrospective nature and heterogeneity of the subjects. One proposed mechanism of the adverse reproductive outcomes in natural and assisted reproduction is impaired sperm chromatin integrity and increased DNA fragmentation rates (52). In a recent systematic review, 17 out of 19 studies demonstrated an association of advanced paternal age with significant increase in DNA fragmentation (53), mostly measured by Sperm Chromatin Structure Assay ^®^ and sperm chromatin dispersion test. The two studies that did not find the effect of advanced paternal age on sperm DNA fragmentation utilized terminal deoxynucleotidyl transferase-mediated deoxyuridine triphosphate nick end labelling (TUNEL) assay. Each sperm chromatin integrity and DNA fragmentation examines different structural aspects of the target molecule with intrinsic advantages and limitations; thus, it is clearly important to use a complementary panel of assays to fully assess sperm quality at the molecular level.

### Impact of Advanced Paternal Age on Offspring Perinatal Health

In a population based cohort study, advanced paternal age was found to increase risk of premature birth, gestational diabetes and newborn seizures (54). The odd ratios of birth defects including cleft lip, diaphragmatic hernia, right ventricular outflow tract obstruction, pulmonary stenosis was found to increase significantly, after adjustment for multiple confounders, with each year of increase in paternal age (55).

### Impact of Advanced Paternal Age on Risks of Malignancy in Offspring

Results from a prospective cohort study of over 180,000 subjects indicate that men >35yrs had a 63% higher risk of having offspring who develop hematologic cancers compared with those whose fathers were <25yr, with a significant linear dose-response association noted (56). In a nationwide cohort study of close to two million children born in Denmark from 1978–2010, the risk of childhood acute lymphoblastic leukemia increases by 13% for every 5 years increase in paternal age (57). Other offspring malignancies associated with advanced paternal age include central nervous system tumors and breast cancer (58–61). One proposed mechanism for increased cancer risk with advanced paternal age is telomeres lengthening (62, 63). Telomere shortening is associated with various diseases and is thought to be a limitation of longevity. Leukocytes telomeres are lengthened in offspring of older fathers by 0.5 -2 times per year of paternal age (62–64). While this may confer some health and longevity advantage, a higher risk for malignancy has been noted (63, 64).

### Impact of Advanced Paternal Age on Risks of Offspring Mental Health

Advanced paternal age is also linked to psychological and neurodevelopmental disorders in offspring (65). The relative risk (RR) of offspring diagnosed with schizophrenia increase progressively with paternal age from 34 years (RR 2.02, 95% CI, 1.17‐3.51 for the 45‐49 age group; RR 2.96, 95% CI, 1.6‐5.47 for the older than 50 group) (66). Other investigators have also reported an increased risk of offspring schizophrenia with advanced paternal age (67–69) unaccounted by other factors such as family history of psychosis, maternal age, parental education and social ability, family social integration, social class, birth order, birth weight or birth complications (70). Additionally, the risk of obsessive-compulsive disorder in offspring was reported to increase with advanced paternal age (71). After adjusting for maternal and family history, the risk of offspring of men >54yrs diagnosed with bipolar disorder was found to be 1.37 times higher than those of men 20–24yrs old (72). Using paternal sibling comparisons, another cohort study reported a 24-fold increase of bipolar disorder in offspring born to fathers 20–24yrs versus those aged 45yrs or older (73). In a population-based cohort study of over 130,000 births, offspring from men aged >40yrs were more than fivefold more likely to develop autism spectrum disorders compared to offspring of men <30yrs (74), consistent with a registry study using paternal sibling comparisons (73).

### Impact of Advanced Paternal Age on Risks of Genetic Disorders in Offspring

Several genetic diseases that occur with a low frequency in the general population are associated with advanced paternal age. These include Apert, Crouzon and Pfeiffer syndromes, achondroplasia and other conditions (75). Many of these disorders follow an autosomal dominant pattern, consistent with the opinion that these are mainly *de novo* mutations in the germline. Although the incidences of these conditions in advanced paternal age are generally lower than 1% (76, 77), they are nonetheless associated with severely debilitating phenotypes. Hence, prospective parents with advanced paternal age concerns should be informed and counselled for such risks.

Approximately 0.33% of infants are born with an altered number of chromosomes. Aneuploidies derive mainly from non-disjunction events during meiotic divisions, represent the most common heritable chromosomal anomaly (78). Though most constitutional aneuploidies originate in the female germline (79), all men produce approximately 3–5% of aneuploidy sperm (80) and non-disjunction events, particularly in sex chromosomes, are more likely to occur with aging (81). Most *de novo* structural chromosomal abnormalities are found to be of paternal origin (82–87). Several studies have shown a significant age related increase in sperm structural chromosomal abnormalities (88–93). Results from studies on the association of advanced paternal age and increased risks of offspring aneuploidies and structural chromosome anomalies are inconsistent (82, 94–101). This is in part related to the fact that the vast majority of chromosome aneuploidies are not compatible with fetal development, leading to implantation failure or early miscarriage. Structural chromosomal rearrangements that are balanced are usually phenotypically normal and are thus undetected during childhood, while the vast majority of those that are unbalanced are not compatible with fetal development.

## Proposed Mechanisms on Advanced Paternal Age Impact

Studies in animal models suggest that the constitution of the male germline is relatively robust with far fewer spontaneous mutations compared to somatic tissues (102, 103). This high level of genetic fidelity in part explains why even after exposure to chemotoxic agents or radiation in men, no increase in the incidence of birth defects, sperm DNA chromatin abnormalities or *de novo* germline mutations are noted in their offspring (104, 105). In contrast, paternal aging has been shown to be unique for the creation of *de novo* mutations in male germline (106). Several mechanisms of age-induced *de novo* germline mutations have been proposed. Cumulative replication error from repeated cell divisions represents a significant source of germline mutation (107, 108). Based on whole-genome sequencing studies of parent-offspring trios, approximately one to three *de novo* mutations are introduced to the germline mutational load of the offspring for each additional year in the father’s age at conception (109, 110). Selfish spermatogonial selection from preferentially amplified mitotic clonal expansion of mutated spermatogonial stem cells (111–113) is another proposed mechanism to explain why several genetic diseases associated with advanced paternal age follow the autosomal dominant pattern. Age-related epigenomic modifications in men, as reported by our group (114) and others (115) are speculated to increase the risk of some rare epigenetic disorders in offspring conceived with ARTs (116). Other proposed mechanisms involve post meiotic damage of sperm DNA secondary to the combined effects of increased oxidative stress (117) and nuclease activities and aberrant or inadequate repair of such damage by oocytes (118).

## Current State of Management of Reproductive Risks Associated With Advanced Paternal Age

Few professional organizations have provided a clear definition of advanced paternal age. The American College of Medical Genetics has defined advanced paternal age as >40yrs at conception (76) for the purpose of risk counselling. While the American Society of Reproductive Medicine states that the sperm donor should be “young enough” (119), the Canadian Fertility and Andrology Society have set an upper age limit for sperm donation at 40yrs (120). However, no organizations have made any clear statements as to whether access to reproductive technologies after this age should be restricted.

The lack of clear, authoritative clinical guidelines not only poses challenges to health providers to decline services, but it also inadvertently allows patients to downplay or ignore the negative impact of paternal aging. Additional factors further aggravate the situation: increased access to contraceptives (121), delayed marriage, high divorce and remarriage rates, increased life-expectancy (122), increased access to erectile dysfunction treatment (123) leading to extension of active sex-life expectancy, continuous spermatogenic activities with aging, social acceptance in delaying fatherhood as modeled by a number of male celebrities having children at advanced age, and widespread usage of social media and dating apps to increase the odds of courtship (124). These factors have provided elements for a perfect storm resulting in a rising number of aging men entering or re-entering fatherhood.

## Challenges in Developing Effective, Practical Strategies to Mitigate the Impact of Advanced Paternal Age

Though experts recognize the importance of disseminating current knowledge on the negative impacts of advanced paternal age to clinicians and prospective parents, in practice, this task is far from simple to execute. For example, when counselling a couple with an aging male partner seeking fertility care, merely informing the couple of the potential adverse outcomes serves little more than risk disclosure. Obviously, the couple could do nothing to change the age factor. Alternative options such as using donor gametes or adoption are unlikely to be accepted when the male partner still has functional sperm. From their perspectives, risk is not a certainty. Infertile couples who are determined enough to pursue fertility treatment may feel entirely rational to accept such risks (125). Additionally, there is ample evidence suggesting that children born to parents of more advanced age may enjoy further benefits in life chances such as financial security, parental psychological maturity and a wider network of support for upbringing, education and future career development (125, 126). Taken together, the impact of counselling solely for risk disclosure may not be effective in modifying behavior or improving treatment outcomes.

To add yet another layer of complexity, denying this couple further fertility evaluation is not correct since there could be significant medical conditions including varicoceles, obstruction of the excurrent ductal system, genetic and endocrinological disorders that can contribute to impaired semen parameters. Some causes of male infertility may be correctable to improve the fertility status of the male partner and allow for a better chance of conception. Further, detection of impaired semen parameters may lead to early detection of potential chronic diseases such as cardiovascular diseases and diabetes mellitus, and even cancers (127–129). It may be unethical not to diagnose and treat their infertility. Even for these couples with no correctable male infertility factors who choose to use ARTs, denying such care based solely on age may be viewed as age-discrimination. Additionally, there is a substantial number of children born to aging fathers from natural pregnancies, yet healthcare providers generally take no action in prohibiting aging men in the society at large to have children. Is it rational for them not to intervene with all men at advanced age who are attempting to have children?

One may propose that a more sensible strategy is perhaps through general public education for a “preventative” approach. Unfortunately, this will also encounter obstacles at a different level. The message that “delayed parenthood could lead to adverse outcomes” may be misinterpreted as “education and career commitment are less important” (130, 131). which would not echo well with the ambitious-minded youngsters Further, as the negative impact of female aging on reproduction risks is arguably stronger than that in male aging (44), if the message is therefore more strongly emphasized to young females than to young males, one could only imagine the severity of backlash it would spark from the public.

With regards to reproductive technologies, though planned or elective egg freezing for non-medical reasons is an established strategy to reduce the reproductive risks associated with female aging, planned or elective sperm freezing has not been shown to be effective in mitigating reproductive and offspring health risks associated with paternal aging. This is in part related to the fact that the well-documented chromatin cryodamage from sperm cryopreservation (132–136) can potentially offset any potential benefits from sperm banking. Though sperm cryopreservation is non-invasive and widely accessible, the fees associated with semen storage for years can be significant. Of note, ARTs are required when using cryopreserved sperm. Intra-uterine insemination (IUI) can be used but given its lower success rate compared to *in vitro* fertilization (IVF) and intracytoplasic sperm injection (ICSI), multiple semen samples may have to be cryopreserved to allow for repeated trials of IUI to have a reasonable success rate. In practice, advanced assisted reproduction such as ICSI are often required when using cryopreserved sperm. Aging men who previously banked sperm at a younger age may opt to attempt conception through intercourse when they realize the cost, invasiveness and potential risks on the female partners and offspring associated with using ICSI (137–139). Ultimately, large scale studies to unbiasedly compare the reproductive outcomes and long-term offspring health of with natural conception versus long-term cryopreserved sperm with ICSI are required to establish the benefits and cost-effectiveness of planned or elective sperm freezing against male aging.

Accumulating evidence from the past two decades links impaired sperm chromatin integrity and DNA fragmentation to increased risk of pregnancy loss and reduced success rate with assisted reproductive technologies. Growing interest in recent years on various sperm selection strategies has led to studies that provided preliminary evidence of improving reproductive outcomes in selected infertile couples (140, 141). However, the question of whether these sperm selection strategies are effective in cases of advanced paternal age, particularly in lowering the risks of health conditions linked to aberrant chromatin, remains to be answered.

## Looking Forward

In dealing with the risks association with advanced paternal age, too often wrong questions were asked: “how old is too old?”, “What is the paternal age cut-off at which we can justify imposing restriction of access to reproductive care?” Although most experts agree that the negative impacts of advanced paternal age can be detected in some men after the age of 40 years, currently there is no consensus on the optimal definition of advanced paternal age as studies have used different age inclusion and different outcomes with different definitions. The progressive nature of the physiological changes associated with male aging is a main reason why it is challenging for investigators to reach agreement on a clear definition for aging.

To begin the mission to reduce risks associated with paternal aging, paradoxically, the focus of discussion must first be shifted away from chronological age to gamete-mediated risk on reproductive outcomes and offspring health. In other words, advanced paternal age should be treated as other male factor infertility causes with a focus on identifying elements that can be ameliorated, assessment of gamete functional status, and selection of the gametes with the best chance for a successful procreation. Health policy makers and healthcare providers may have to accept the fact that the growing number of aging men having children is an inevitable phenomenon in the current direction of societal evolution. It is equally important to recognize that strategies aiming to prohibit or dissuade this behavior through establishing a clear paternal age limit for provision of fertility care or through education and counselling can readily be challenged and therefore deemed ineffective.

An alternative approach is to have policy makers, clinicians and investigators work closely together to synthesize information on the risks that can be disseminated to prospective parents to allow them to engage in a shared decision-making model with their healthcare providers. Risks on adverse reproductive outcomes and offspring health that are gamete mediated should be comprehensively assessed and defined, using established diagnostic tools at the molecular levels. It is important to emphasize that, in addition to aging, gamete mediated risks may well be attributed to other health conditions such as intrinsic genetic disorders, gonadotoxin exposure, history of cytotoxic therapies, metabolic derangements, obesity, smoking, and varicoceles. Thus, communication of gamete mediated reproductive risks should be conducted across the board as a standard of practice to all male partners seeking fertility care and not just to those at an advanced age. Shifting the focus of counselling from chronological age to gamete mediated risks allows clinicians to formulate a treatment plan or decline treatment without being accused of age discrimination. Finally, additional psychosocial concerns beyond gamete quality in the context of advanced paternal age such as life-expectancy of parents, should also be an important consideration in this shared decision-making model.

To minimize or mitigate the negative impact of advanced paternal age, comprehension of the collective body of scientific evidence is only the first step. Continued dialogues must be maintained among stakeholders at all levels, including investigators, healthcare providers, health policy makers and patients, on emerging data and their implications at the personal as well as societal levels. Most importantly, it is imperative for all parties to collaborate rigorously, with the goal of catalyzing a new agenda to reconceptualize the management strategy of advanced paternal age in the context of reproductive care of prospective parents.

## Author Contributions

PC and BR contributed to the writing and the editing of this manuscript. All authors contributed to the article and approved the submitted version.

## Funding

This research has been funded by the CIHR Institute for Gender and Health Team Grant TE1-138298. BR is a James McGill Professor.

## Conflict of Interest

The authors declare that the research was conducted in the absence of any commercial or financial relationships that could be construed as a potential conflict of interest.

## Publisher’s Note

All claims expressed in this article are solely those of the authors and do not necessarily represent those of their affiliated organizations, or those of the publisher, the editors and the reviewers. Any product that may be evaluated in this article, or claim that may be made by its manufacturer, is not guaranteed or endorsed by the publisher.
